# Electron and lattice dynamics of transition metal thin films observed by ultrafast electron diffraction and transient optical measurements

**DOI:** 10.1063/1.4971210

**Published:** 2016-12-07

**Authors:** A. Nakamura, T. Shimojima, M. Nakano, Y. Iwasa, K. Ishizaka

**Affiliations:** Quantum-Phase Electronics Center and Department of Applied Physics, The University of Tokyo, Bunkyo, Tokyo 113-8656, Japan

## Abstract

We report the ultrafast dynamics of electrons and lattice in transition metal thin films (Au, Cu, and Mo) investigated by a combination of ultrafast electron diffraction (UED) and pump-probe optical methods. For a single-crystalline Au thin film, we observe the suppression of the diffraction intensity occuring in 10 ps, which direcly reflects the lattice thermalization via the electron-phonon interaction. By using the two-temperature model, the electron-phonon coupling constant (*g*) and the electron and lattice temperatures (*T*_e_, *T*_l_) are evaluated from UED, with which we simulate the transient optical transmittance. The simulation well agrees with the experimentally obtained transmittance data, except for the slight deviations at the initial photoexcitation and the relaxed quasi-equilibrium state. We also present the results similarly obtained for polycrystalline Au, Cu, and Mo thin films and demonstrate the electron and lattice dynamics occurring in metals with different electron-phonon coupling strengths.

## I. INTRODUCTION

Investigation of the nonequilibrium states in solids induced by the photoexcitation has been one of the most challenging problems in condensed matter physics. In some simple systems such as noble metals, a common approach to describing the electron and lattice dynamics is the so-called two-temperature model, which assumes that electrons and phonons are in thermal quasi-equilibriums with two different temperatures. When a solid is irradiated by a femtosecond laser pulse, the electrons are immediately (non-thermally) excited and quickly transferred into deeper parts of the sample.^1^ The electron subsystem is then quasi-thermalized via electron-electron interactions and accordingly starts to follow the Fermi-Dirac statistics characterized by the electron temperature (*T*_e_). The excess energy in the electron subsystem is redistributed to the lattice through the electron-phonon interactions usually within a few picoseconds, thus raising the lattice temperature (*T*_l_).^2,3^ The two-temperature model was originally proposed by Anisimov *et al*.^4^ The time-dependent relation between *T*_e_ and *T*_l_ can be written in the form

(1) $$\gamma T_{e}\frac{\partial T_{e}(t)}{\partial t}=-g(T_{e}-T_{1})+P(t),$$ (1a)

$$C_{1}\frac{\partial T_{1}(t)}{\partial t}=g(T_{e}-T_{1}),$$ (1b)

where *γ*, *C*_l_, and *P*(*t*) are the electronic specific heat coefficient, the lattice heat capacity, and the absorbed laser power, respectively, per unit volume.

Over the last couple of decades, transient optical reflectivity measurements that are sensitive to *T*_e_ have significantly contributed to the estimation of *T*_e_, *T*_l_, and *g* values by two-temperature model analysis in metals and superconducting materials.^2,5^ More recently, much more sophisticated models beyond these two temperatures have been suggested to discuss the many-body interaction residing in complex materials such as cuprate superconductors.^6,7^ Thus, the importance of the reliable analysis of transient temperatures in the out-of-equilibrium state has been rapidly increasing from the viewpoint of materials science. However, the quantitative evaluation of *T*_e_ and *T*_l_ is still difficult from an optical measurement alone, since it usually lacks the information of lattice. The accurate derivation of *T*_e_ is also not so simple, as the reflectivity or transmittance usually is a complicated function of temperature. Thus, the experimental evaluation of *T*_e_ and *T*_l_ by using various kinds of probes beyond optical methods has been long desired.

Time-resolved photoemission spectroscopy and diffraction measurements are the promising methods to detect *T*_e_ and *T*_l_ without ambiguity. Time-resolved photoemission spectroscopy can directly obtain *T*_e_ by analyzing the cutoff of the photoemission spectra at the Fermi level.^1,8^ On the other hand, diffraction measurements can detect *T*_l_ straightforwardly through the Debye-Waller effect, by recording the transient diffraction intensity. Recently, the ultrafast electron diffraction (UED)^9,10^ and the x-ray diffraction (XRD)^11–13^ techniques have been developed for investigating the transient lattice dynamics of materials. In the weak excitation regime, *g* values of noble metals such as Au and Ag have been estimated from the UED data^14^ by using two-temperature model analysis. For cuprate superconductors, three temperature analysis has been performed and its comparison with the photoemission spectroscopy is also reported.^15^ On the other hand, under the extremely strong laser excitation, the mechanism of the ultrafast melting of Au and Al nanofilms has also been investigated by UED and the ultrafast x-ray diffraction.^16–19^ While the x-ray diffraction method has a much higher momentum resolution, UED is a suitable technique for effectively evaluating the lattice dynamics of thin films of nanometer thickness, because of the larger scattering cross sections of electrons as compared to the x-ray.

In this paper, we report the lattice and electron dynamics of thin films of elemental metals by using UED and transient optical measurements. Since the processes of thermalization and relaxation strongly depend on the sample configuration, especially the thickness of the films,^17^ the same samples are used for both the probes. For the single crystalline Au thin film, the UED and the optical transmittance data are successfully analyzed by the common two temperature model analysis. The slight inconsistency between the obtained transient transmittance and UED results is found, which may be partly arising from the difficulty of simulating the transmittance data probed at the wavelength very close to the interband transition. We also measure the polycrystalline Au, Cu, and Mo thin films by the combination of UED and optical measurements and demonstrate the lattice and electron dynamics in compounds with different electron-phonon couplings.

## II. METHODS

**FIG. 1.**
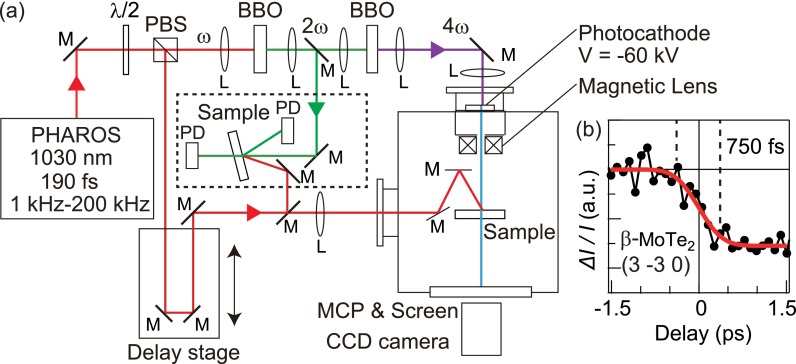
(a) Schematic experimental setups for UED and transient optical reflectivity measurements. The fundamental laser pulse (1.2 eV) is split into two branches. One is passed through a delay line for exciting the sample. The other part is utilized to generate the frequency quadrupled ultraviolet pulse (4.8 eV) for driving the electron gun. Electron pulses are emitted from a photocathode and accelerated to 60 keV. Electron diffraction patterns are obtained using a phosphor screen, MCP, and CCD camera. (b) The time evolution of the (3 −3 0) diffraction intensity of β-MoTe_2_ is indicated by the black markers. The solid red curve represents the fitting by Gaussian cumulative distribution function. The evaluated upper limit of the total time resolution is 750 ± 200 fs.

A schematic of the experimental setup for UED is shown in Fig. 1(a). It consists of the femtosecond (190 fs) laser system (PHAROS, Light Conversion) and the ultrahigh-vacuum (∼10^−10^ Torr) chamber for the diffraction measurement. The repetition rate of the laser can be changed from 1 kHz to 200 kHz. The generated laser is split into two beams, pump and probe, by a polarized beam splitter (PBS). The fundamental 1030 nm photon pulse for pumping passes through a delay line and is used to excite the sample. The rest of the beam passes through two *β*-Ba_2_B_2_O_4_ (BBO) crystals for the fourth harmonic generation. The frequency quadrupled 257 nm photon pulse is then focused on the photocathode made of a 10 nm-thick Au film where the electron packet for the probe is generated via the photoemission process. The generated electron packets are accelerated to 60 keV and focused onto the sample by a magnetic lens. The pump laser spot diameter, 300 *μ*m, is chosen to be sufficiently larger than that of the probe electron beam (120 *μ*m) to ensure that the homogeneously excited region of the sample is probed. The overlap of the pump and probe beams is aligned by passing them through a pinhole of 50 *μ*m diameter. In the transmission geometry, the diffracted electrons are recorded by using a phosphor screen with a microchannel plate detector (MCP) and a charge-coupled device (CCD) camera. The rough estimation of the total time resolution of the system *Δt*_total_ is done by measuring the time evolution of the diffraction intensity of the reference sample that shows a rapid response (*β*-MoTe_2_), as shown in Fig. 1(b). The solid red curve indicates the best result of fitting by the Gaussian cumulative distribution function, which gives the evaluated upper limit of *Δt*_total_ = 750 ± 200 fs.

Transient optical reflectivity and transmittance measurements were also performed using the same optical setup, to confirm the behavior of *T*_e_. We note that it is important to use the same samples for both measurements of *T*_e_ and *T*_l_ since the time-scale of the energy relaxation can sometimes strongly depend on the film thickness.^20^ As shown in Fig. 1(a), the frequency doubled 515 nm photon pulse is used to probe the sample, which is photoexcited by the 1030 nm photon pulse. The reflectivity and transmittance were measured by using a photo detector (PD). The time resolutions of the optical reflectivity and transmittance measurements are estimated to be 280 fs and 550 fs, respectively.

A free-standing single-crystalline Au film of 11 nm thickness (Oken Shoji) fixed on a copper microgrid was used for UED and optical transmittance measurements. Polycrystalline films of Au, Cu, and Mo with the thickness of 5 nm were prepared *ex-situ* by the electron beam physical vapor deposition method. The films were deposited onto the copper microgrids covered with carbon membranes for UED measurements, whereas thin glass substrates were used for optical reflectivity measurements. The deposition rate was set to 0.1 Å/s and the base pressure of the chamber was in the order of 10^−7 ^Pa. Thus, obtained films were uniformly distributed on the substrates, as confirmed by the scanning electron microscope. The wave length of the pump light, 1030 nm, is within the plasma edge of the all samples. For simplicity, we thus assume that the photoexcitation by pumping is dominated by the intraband transition, for applying the two-temperature model.^7^

## III. RESULTS AND DISCUSSIONS

**FIG. 2.**
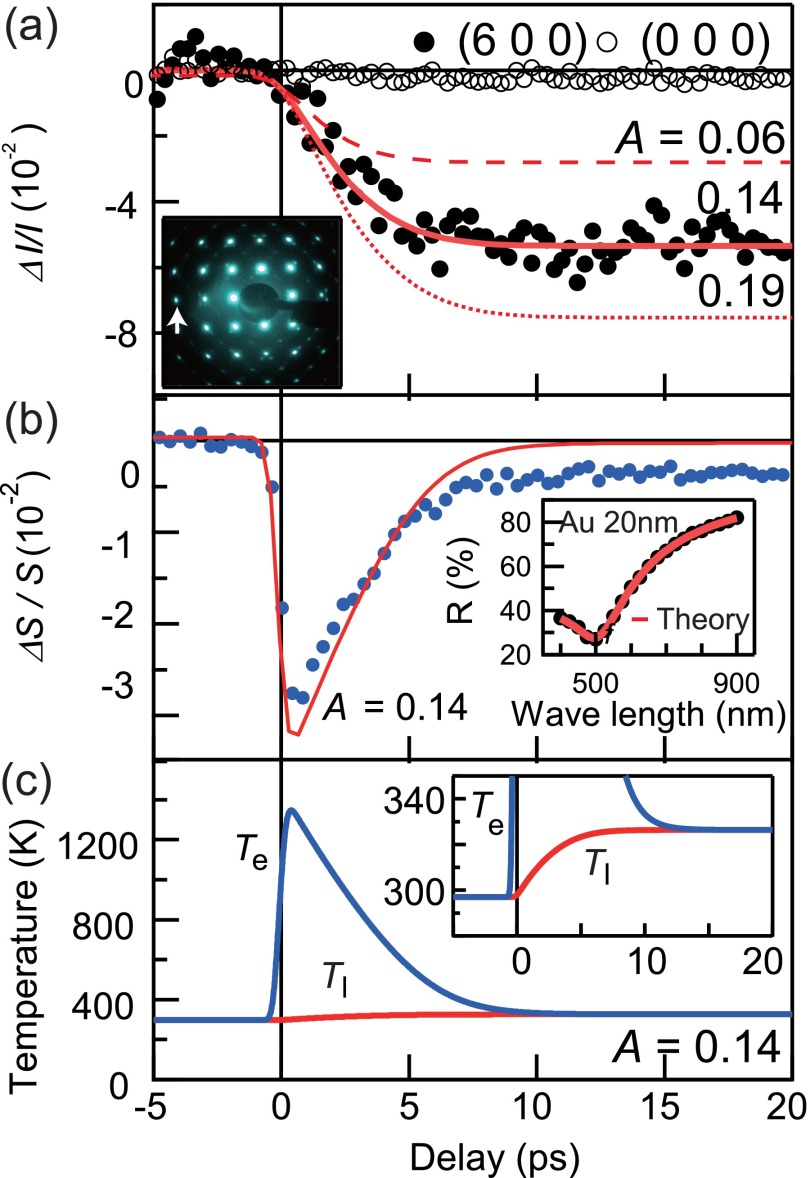
(a) The relative intensity change of the (600) Bragg spot for a single-crystalline Au film of 11 nm thickness obtained at a repetition rate of 10 kHz with a fluence of 0.8 mJ/cm^2^. The solid red curve represents the best fitting result by the two-temperature model analysis, where the evaluated values of *g* and *A* are 1.8 ± 0.5 (10^16 ^W/m^3^ K) and 0.14 ± 0.02, respectively. Those with *A* = 0.06 and 0.19 are also displayed as the references shown by the broken curves. The inset shows the static electron diffraction pattern. The diffraction pattern was obtained at room temperature with the repetition rate of 200 kHz. The white arrow indicates the diffraction spot (6 0 0) used for the analysis. (b) The transient transmittance data for 11 nm single-crystalline gold. The solid red curve shows the simulated transmittance from *T*_e_ in Fig. 2(c). The inset shows the static reflectivity spectrum of the 20 nm gold film in the literature.^31^ The solid red curve represents the theoretical fitting described in a previous study.^29^ (c) The calculated *T*_e_ and *T*_l_ with *g* = 1.8 (10^16 ^W/m^3^ K) and *A* = 0.14. Other fixed parameters are listed in Table I. Both *T*_e_ and *T*_l_ reach the quasi-equilibrium temperature 326 K at ∼10 ps after photoexcitation.

We begin with the UED results obtained for the single-crystalline Au with a thickness of 11 nm to quantitatively determine the transient *T*_l_ and the value of *g*. The static electron diffraction pattern was obtained at room temperature (RT) with a repetition rate of 200 kHz as shown in the inset of Fig. 2(a). The intense Bragg peaks indicate the reciprocal lattice of the face-centered cubic (fcc) structure. Figure 2(a) shows the time evolution of the relative (600)-peak intensity, *ΔI*(*t*)/*I* = [*I*(*t*) – *I*(*t* < 0)]/*I*(*t* < 0), where *I*(*t*) and *I*(*t* < 0) are the integrated intensities of the (600) peak at time *t* and *t* < 0 (before the photoexcitation), respectively. They are obtained at a repetition rate of 10 kHz under the pump fluence of 0.8 mJ/cm^2^. The (600)-peak intensity is suppressed by ∼5% within ∼10 ps. We note that this time scale is in good agreement with previous UED studies on thin films^14,16,19^ and nano particles^21^ of gold, which are in the range of 10–40 ps depending on the sample thickness, shape, and excitation fluences. In this weak excitation regime, the suppression of the Bragg peak intensity can be regarded as the signature of the lattice thermalization and expressed by using the Debye-Waller-factor temperature parameter *Y*(*T*_l_)^22^ as

$$\;{\text{log}}_{10}(1+\Delta I/I)={\left(\text{sin}\;\theta /L\right)}^{2}Y(T_{1}),$$ (2)

where *L* is the wave length of the probe electron beam (0.491 pm) and *θ* is the Bragg angle. Within the high-temperature limit Debye model, *Y*(*T*_l_) is expressed as

$$Y(T_{1})=-(\;{\text{log}}_{10}e)\frac{48\pi^{2}\hbar^{2}}{Mk_{B}{\Theta_{D}}^{2}}(T_{1}-T_{0}),$$ (3)

where ℏ is the reduced Planck constant, *k*_B_ is the Boltzmann constant, *T*_0_ is the original lattice temperature before the photoexcitation (Δ*I* = 0), and *Θ*_D_ is the Debye temperature. Applying Eqs. (2) and (3), Δ*I*(*t*)/*I* can be directly related to the transient lattice temperature *T*_l_(*t*). The Debye-Waller coefficient d*Y*/d*T*_l_ in Eq. (3) is evaluated from static x-ray diffraction measurements at several temperatures and theoretical calculations.^22,23^ The value of d*Y*/d*T*_l_ we used for Au is −1.9 × 10^−3^ Å^2^/K, which corresponds to *Θ*_D_ = 157 K.

For the estimation of *g* and *T*_l_, Δ*I*(*t*)/*I* curves were fitted by using the *T*_l_(*t*) solutions of the two-temperature model [Eq. (1)] convolved with a Gaussian including the total time resolution of the system (*Δt*_total_ = 750 fs). The source term *P*(*t*) representing the volume-averaged absorbed pump laser power in Eq. (1) is given by

P(t)=AF(t)/d, (4)

where *d* is the film thickness and *F*(*t*) is the pump laser flux per unit area which is a Gaussian function of *t* with the width of *Δt*_pump_ = 190 fs. *A* is expressed as *A* = 1 – *R* – *S*, where *R* and *S* are the reflectivity and transmittance of the thin-film sample, respectively. Since the film thickness of 11 nm is much shorter than the ballistic range of the hot electrons in typical metals (20–100 nm),^20^ we do not need to consider the dissipation process of the hot electrons into the deeper part of the sample. We note that the multiple scattering effect is negligible in our experimental condition.^24^ It has also been pointed out that *g* and the electronic heat capacity can be a non-monotonic function of *T*_e_, depending on the shape of the density of states near the Fermi level.^7,16,25,26^ However, our experimental condition that raises *T*_e_ to ∼1300 K at the highest should allow us to use the constant *g* and *γ* values in Eq. (1a).^25,26^ We also assume the constant *C*_l_ since the temperature of the measurement is quite high as compared to the Debye temperature (*Θ*_D_ = 157 K).

For the analysis, we set *g* and *A* as the fitting parameters, whereas *dY/dT*_l_ and *γ* are fixed as given in Table I. The *C*_l_ values are calculated by using the Dulong-Petit law for all samples (*C*_l_ = 25 J K^−1 ^mol^−1^). We note that one can obtain a unique pair of *g* and *A* values since the former is independently related to the time scale of the *ΔI*/*I* curve while the latter mainly affects the height of *ΔI*/*I.* This tendency is evidenced in the three curves with a fixed *g* value [*g* = 1.8 ± 0.5 (10^16 ^W/m^3^ K)] and three different *A* values (0.06, 0.14, and 0.19) in Fig. 2(a). The solid red curve in Fig. 2(a) represents the best fitting result with *A* = 0.14 ± 0.02. The value of *A* should naturally depend on the detailed form (especially the thickness) of the thin-film sample but is consistent with the results reported so far.^27^ *T*_e_ and *T*_l_ thus obtained by the two-temperature model analysis are displayed in Fig. 2(c). *T*_l_ increases monotonically from RT to the quasi-equilibrium temperature (*T*_q_) of 326 ± 5 K. On the other hand, after the rapid increase of *T*_e_ to ∼1300 K immediately after the photoexcitation, *T*_e_ decreases down to *T*_q_ in ∼10 ps. In previous studies, the time evolution of *T*_e_ for Au was obtained through the two temperature model analysis of the pump-probe optical reflectivity measurements.^20,28^ In Ref. 20, the transient reflectivity for the Au film of 20 nm thickness shows the relaxation of *T*_e_ within about 10 ps and the obtained *g* value is 2.1 ± 0.3 (10^16 ^W/m^3^ K), which are more or less consistent with our results.

**TABLE I.** The values of the electronic specific heat coefficient *γ* and the Debye-Waller factor coefficient d*Y*/d*T*_l_ used for the two-temperature model fitting analysis, and the electron-phonon coupling constant *g* obtained in the present work. *γ* and d*Y*/d*T*_l_ are taken from Refs. 22, 23, 35, and 36.

	*γ* (J/m^3^ K^2^)^29^	d*Y*/d*T*_l_ (10^−3^Å^2^/K^2^)^17,18,28^	*g* (10^16 ^W/m^3^ K) Present work
Au	71	−1.9	1.2 ± 0.5 (1.8 ± 0.5 for single crystal)
Cu	98	−2.0	25 ± 5
Mo	211	−0.5	40 ± 10

To more quantitatively discuss the transient behaviors of *T*_e_ and *T*_l_, we also measured the transient transmittance of the identical sample under the same experimental conditions. The time evolution of the relative transmittance, *ΔS*(*t*)/*S* = [*S*(*t*) − *S*(*t* < 0)]/*S*(*t* < 0), probed by 515 nm light is indicated by the solid blue markers in Fig. 2(b). For comparison, the transmittance simulated from the *T*_e_ obtained by the two temperature analysis on UED [Fig. 2(c)] is also presented by the red curve. This simulated curve is derived from the model described in the literature,^29^ which considers the plasmonic response and the *d-p* interband transition of electrons. Here, the electron temperature dependence of the dielectric function *ε*(*T*_e_,*ω*) is given by

$$\varepsilon (T_{e},\omega )=1-\frac{\omega_{p}^{2}}{\omega (\omega +i/\tau )}+3\frac{\omega_{p}^{2}}{\omega^{2}}\frac{f_{d}{\;}_{p}}{k_{F}(T_{e})E_{F}(T_{e})}\int_{0}^{\sqrt[{3}]{2k_{F}}}\left(\frac{z_{1}^{2}}{k^{2}-z_{1}^{2}}-\frac{{z^{\prime }}_{1}^{2}}{k^{2}+{z^{\prime }}_{1}^{2}}-\frac{2z_{0}^{2}}{k^{2}-z_{0}^{2}}\right)E_{pk}[1-n(T_{e},k)]dk,$$ (5)

with the abbreviations

$$\begin{array}{c} z_{n}^{2}=\frac{2m}{\hbar^{2}}(n\hbar \omega +in\hbar \omega_{c}-\Delta ),\\ z_{n}^{\prime 2}=\frac{2m}{\hbar^{2}}(n\hbar \omega +in\hbar \omega_{c}+\Delta ),\\ E_{p}{\;}_{k}=\Delta +\frac{k^{2}\hbar^{2}}{2m}\\ E_{F}(T_{e})=\frac{\hbar^{2}k_{F}^{2}(T_{e})}{2m}=E_{F0}\left[1-\frac{\pi^{2}}{12}{\left(\frac{k_{B}T_{e}}{E_{F}{\;}_{0}}\right)}^{2}\right]\\ \tau =\tau_{0}{\left(\frac{E_{F}}{\hbar \omega -E_{F}}\right)}^{2}, \end{array}$$ (6)

where *n*(*T*_e_,*k*) is the Fermi-Dirac distribution function at the energy of *ℏ*^2^*k*^2^/2*m*. *ω_p_*, *E*_F0_, and *τ*_0_ are the plasma frequency, Fermi energy, and the relaxation time of electrons, respectively. The parameters *Δ*, *f*_dp_, *ω*_c_ are the energy gap between the *d* and *p* bands at the center of the Brillouin zone, the oscillator strength for *d-p* transitions, and the electron collision frequency. The first two terms in Eq. (5) describe the plasma (or intraband) contribution of the dielectric function while the last term denotes the interband transition near 2.4 eV. We used literature values^30^ for *ω*_p_ = 8.45 eV, *E*_F0_ = 5.53 eV, and *τ*_0_ = 1.4 × 10^−14^ s and determined *Δ* = −2.99 eV, *f*_dp_ = 0.407, and *ω*_c_ = 0.273 eV by fitting Eq. (5) to the experimental reflectivity spectrum of the 20 nm Au film in the literature^31^ as shown in the Fig. 2(b) inset. The *ΔS*/*S* thus obtained from *T*_e_(*t*) shows a good agreement with the experimental data in Fig. 2(b), except for the slight deviations at the initial photoexcitation and the relaxed quasi-equilibrium state. The relaxation process within the 10 ps corresponds to the rising time of the UED in Fig. 2(a), providing the time scale where the electron and lattice subsystems attain the common quasi-equilibrium state (*T*_e_ ≈ *T*_l_ ≈ *T*_q_). This result ensures that the electron-phonon coupling *g* can be safely determined by the two-temperature model analysis on either UED result or optical transmittance measurements. On the other hand, when we closely focus on the *ΔS*/*S* curves, the quantitative agreement between the simulation and the experiment is not perfectly achieved. This may be due to the complicated behavior of *ΔS*/*S* probed by 515 nm light, the wavelength very close to the interband transition of Au. Since the *ε*(*T*_e_,*ω*) model (Eqs. (5) and (6)) assumes the simple spherical Fermi surface and also the temperature dependence is taken into account only for the electron distributions, more realistic calculations of the temperature-dependent electronic structures may be necessary to fully obtain the quantitative correspondence between *T*_e_ and *ΔS*/*S*, especially in the high-*T*_e_ region (<2 ps). The mismatch of *ΔS*/*S* at the quasi-equilibrium state (>10 ps) has been often attributed to the strain of the lattice derived from lattice heating, which is not included in the present two-temperature model analysis.^3,28^ According to the previous x-ray diffraction study,^32^ the thermal expansion of the lattice constant in this region (*T*_q_ ≈ 326 K) may reach 0.25%. Unfortunately, this expansion is far below the momentum resolution of our UED measurement to be detected. More quantitative evaluation will be available by utilizing the complementary experiments of UED, XRD, and optical probes in future.

**FIG. 3.**
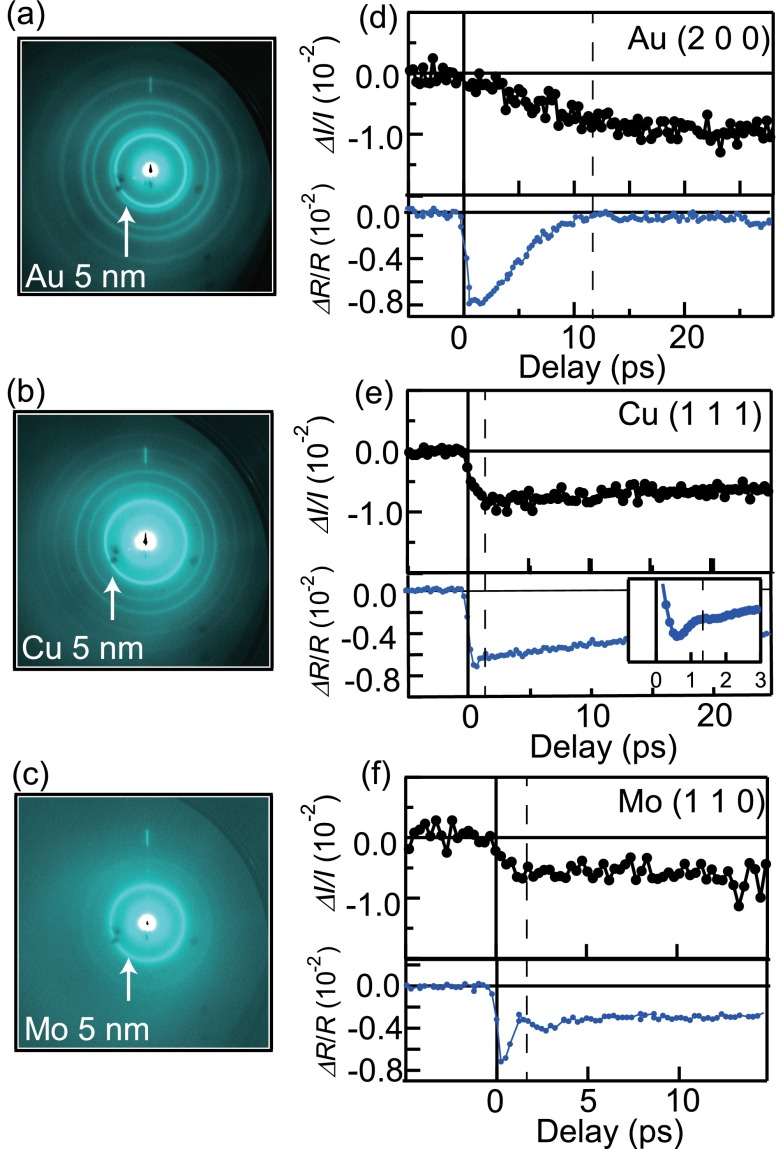
(a)–(c) The static electron diffraction patterns for polycrystalline 5-nm-thick films of Au, Cu, and Mo, respectively. These diffraction patterns were obtained at room temperature with the repetition rate of 200 kHz. White arrows indicate the diffraction rings used for the analysis. (d)–(f) UED and the transient optical reflectivity data for polycrystalline Au, Cu, and Mo, respectively. These data were obtained at a repetition rate of 10 kHz with a fluence of 0.95 mJ/cm^2^. The broken lines represent the timescales of the lattice thermalization.

Now we apply the similar measurements and analysis to other elemental metals. Figures 3(a)–3(c) show the static electron diffraction images for polycrystalline 5 nm thin films of Au, Cu, and Mo, respectively. The diffraction rings were identified to be an fcc structure for Au and Cu, and body-centered cubic (bcc) structure for Mo. *ΔI*/*I* of particular indices, obtained by integrating the intensity of each ring, is presented by the black markers in the upper panels of Figs. 3(d)–3(f). The *ΔI*/*I* curves exhibit the rapid suppression of Bragg intensities after the photoexcitation with different time scales, depending on the element.

By looking at the UED and transient optical reflectivity data as shown in upper and lower panels in Figs. 3(d)–3(f), respectively, we can discuss the time evolution of *T*_e_ and *T*_l_. The transient optical reflectivity, being sensitive to the change in *T*_e_, exhibits a relaxation time that is comparable to the rise time of *ΔI*/*I*, as indicated by the dotted lines in Figs. 3(d)–3(f), where *ΔR*(*t*)/*R* denotes [*R*(*t*) – *R*(*t* < 0)]/*R*(*t* < 0). It is clearly shown that the lattice thermalization due to the energy transfer from the electron to the lattice subsystems occurs in Au more slowly than that in Cu and Mo, suggestive of the weaker electron-phonon coupling for Au. Once the quasi-equilibrium state is attained, *ΔI*/*I* and *ΔR*/*R* show the similar long-term time dependence, indicating that the relation *T*_e_ ≈ *T*_l_ ≈ *T*_q_ holds thereafter. The slow relaxation of *T*_q_ in this regime should be reflecting the gradual cooling of the local temperature through the heat diffusion. This long-term relaxation process seems to be somewhat faster for Cu (∼60 ps) as compared to Au and Mo (>100 ps). It should be reflecting the difference of the thermal conductivity (Cu: ∼400 W/mK, Au: 318 W/mK, and Mo: 138 W/mK near RT).

**FIG. 4.**
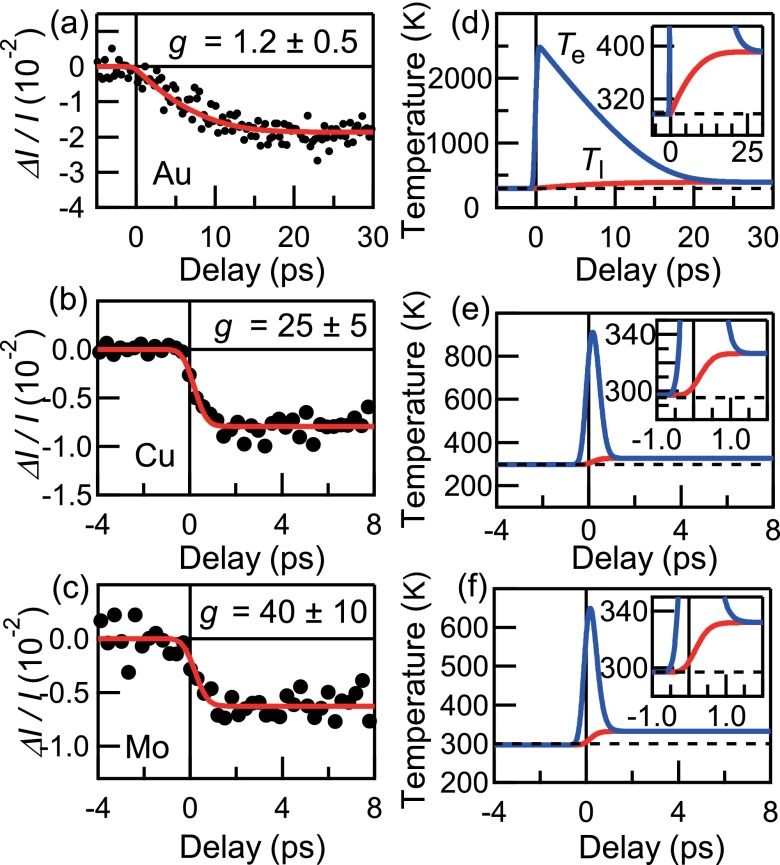
(a)–(c) UED data with respective fitting curves for polycrystalline 5-nm-thick films of Au, Cu, and Mo, respectively. Red solid curves represent the best fit obtained by the two-temperature model analysis with the *g* values of 1.2, 25, and 40 × 10^16 ^W/m^3^ K, respectively. The calculated *T*_e_ and *T*_l_ for Au (d), Cu (e), and Mo (f) with the g values obtained from the two-temperature model analysis.

To obtain the *T*_l_ and *g* values, *ΔI*/*I* curves were analyzed using the two-temperature model as in the case for the single-crystalline Au. We again assume the temperature-independent *γ*, *C*_l_, and *g* for these analyses, since they will be least affected in the present range of *T*_e_. The fixed parameters are summarized in Table I. As shown in Figs. 4(a)–4(c), *ΔI*/*I* curves for Au, Cu, and Mo were well reproduced by the fitting curves represented by the red solid curves. The *T*_e_ and *T*_l_ used in the analysis are also displayed in Figs. 4(d)–4(f). The obtained *g* value for Au, 1.2 ± 0.5 × 10^16 ^W/m^3^ K, agrees well with the result on the 11 nm single-crystalline sample and is found to be much smaller than those for Cu (25 ± 5 × 10^16 ^W/m^3^ K) and Mo (40 ± 10 × 10^16 ^W/m^3^ K). Reflecting this difference, the behaviors of *T*_e_ and *T*_l_ are also strongly element-dependent. While the *T*_e_ of Au increases up to >2000 K, those for Cu (Mo) are at most about 900 K (700 K) and quickly cool down to *T*_q_ via the electron-phonon coupling. Here, we note that the *T*_e_ curve for Au is very similar to *ΔR*/*R* in Fig. 3(d). It suggests that *ΔR*/*R* at 515 nm shows a fairly monotonic dependence on *T*_e_ in the case of Au. For Mo and Cu, in contrast, *ΔR*/*R* seems to be saturated when *T*_e_ gets higher beyond about 400 K. Such non-monotonic temperature dependence of the reflectivity has often been reported,^20^ which makes the two-temperature analysis very complicated. In the present study, the Debye-Waller analysis of the diffracted intensity significantly helps the quantitative discussion of the two-temperature model.

Here, we note that the *g* values for these metals calculated by using the linear response method^33^ give a similar tendency with our result. The *g* values for various cubic metals have also been previously evaluated by using the transient optical reflectivity measurements.^2,5,20,34^ Regarding Au, there are several studies (see Ref. 20 and references therein) reporting the *g* values of ∼2 × 10^16 ^W/m^3^ K, which are quantitatively consistent with the present UED result. The *g* values reported for Cu (10 × 10^16 ^W/m^3^ K)^2^ and Mo (13 × 10^16 ^W/m^3^ K)^34^ are again much larger than that of Au, giving the qualitative similarity with UED. Quantitatively, however, they are somewhat smaller compared to the present work. It may be at least partly due to the difficulty of the *T*_e_ estimation from the optical reflectivity in the strong electron-phonon coupled system.

## IV. CONCLUSION

In conclusion, we investigated the ultrafast dynamics of electrons and lattice in transition metal thin films (Au, Cu, and Mo) by a combination of ultrafast electron diffraction (UED) and pump-probe optical methods. For a single-crystalline Au thin film (11 nm), we observed the suppression of the diffraction intensity occuring in 10 ps, which was analyzed by using the two temperature model and the Debye-Waller factor. The electron-phonon coupling constant (*g*) and the electron and lattice temperatures (*T*_e_, *T*_l_) evaluated from UED were used to simulate the transient optical transmittance, which showed a good agreement with the experiment. The slight deviations observed at the initial photoexcitation and the relaxed quasi-equilibrium state could be due to the insufficiency in analyzing the temperature-dependent dielectric function that was used in deriving the transmittance. The results for polycrystalline Au, Cu, and Mo thin films (5 nm) were also presented, and the electron and lattice dynamics occurring in metals with different electron-phonon coupling strengths were clearly demonstrated. UED will be also useful for quantitatively investigating the out-of-equilibrium states in a variety of complex materials, such as unconventional superconductors, strongly correlated systems, photocatalytic nanoparticles, and so on.
